# Robot-assisted laparoscopic combined with endoscopic partial gastrectomy (RALE-PG) for the treatment of gastric gastrointestinal stromal tumors in challenging anatomical locations: single-center experience

**DOI:** 10.3389/fsurg.2024.1391387

**Published:** 2024-05-23

**Authors:** Chenxing Jian, Xinxiang Huang, Ruirong Lin, Weijin Yang, Shiyao Zheng, Hongxin He, Shangkun Jin, Chunkang Yang, Shen Guan

**Affiliations:** ^1^ Department of Colorectal Surgery, Clinical Oncology School of Fujian Medical University, Fujian Cancer Hospital, Fuzhou, China; ^2^ School of Clinical Medicine, Fujian Medical University, Fuzhou, China; ^3^ Department of Anorectal Surgery, Affiliated Hospital of Putian University, Putian, China

**Keywords:** gastrointestinal stromal tumors, challenging anatomical location, endoscopic partial gastrectomy, manual suturing, robot-assisted laparoscopic surgery

## Abstract

**Background:**

Gastric gastrointestinal stromal tumors in challenging anatomical locations are difficult to remove.

**Methods:**

This study retrospectively analyzed the clinical data of 12 patients with gastric GISTs in challenging anatomical locations who underwent robot-assisted laparoscopic combined with endoscopic partial gastrectomy (RALE-PG) and manual suturing of the gastric wall.

**Results:**

This study included 12 patients with a mean age of 56.8 ± 9.8 years and a mean BMI of 23.9 ± 1.9 kg/m^2^. Tumors were located in the GEJ (*n* = 3), lesser curvature (*n* = 3), posterior gastric wall (*n* = 3) and antrum (*n* = 3). The cardia and pylorus were successfully preserved in all patients regardless of the tumor location. The mean tumor size was 4.5 ± 1.4 cm. The mitotic-count/50 mm^2^ was less than 5 in all patients (100%). There was no intraoperative tumor rupture (0%) and no conversion to open surgery (0%). The median operation time was 122 (97–240) min, and the median blood loss volume was 10 (5–30) ml. The median postoperative VAS score was 2 (2–4). The median time to first flatus was 2 (2–3) days. The median time to first fluid intake was 2 (2–3) days. The median time to first ambulation after the operation was 3 (2–4) days. No cases of anastomotic stenosis or leakage were found. The median time to drain removal for 6 patients was 5 (4–7) days. The median time to nasogastric tube removal for all patients was 2 (1–5) days. The median postoperative hospital stay was 5 (4–8) days. One patient (female/41 year) developed moderate anemia (Clavien-Dindo grade II complication). There was no unplanned readmission within 30 days after the operation. The median distance from the tumor to the resection margin was 1 (1–2) cm. R0 resection was achieved in all patients. The median follow-up period was 19 (10–25) months, and all patients survived with no recurrence or metastasis.

**Conclusions:**

RALE-PG is a safe, feasible and advantageous technique for treating GISTs in challenging anatomical locations. It can be used to accurately remove the tumor while preserving gastric function to the greatest extent, but long-term oncologic outcomes need to be evaluated in a study with a larger sample size and longer follow-up period.

## Introduction

A gastrointestinal stromal tumor (GIST) is a common mesenchymal tumor (1), with an annual incidence of 1–2 per 100,000 people (2). GISTs can be found in any part of the digestive tract, including the stomach (40%–50%) and small intestine (20%–40%) (3). Surgical removal is the preferred treatment for gastric stromal tumors (4), and negative surgical margins of pathological specimens are considered a mandatory requirement (5). Due to the low rate of lymph node metastasis, routine prophylactic lymph node dissection is not recommended (6, 7).

In 2000, Kimata et al. (8) reported the first laparoscopic surgery for GISTs. Since then, laparoscopic surgery has been widely accepted for the treatment of GISTs. Many retrospective studies have demonstrated the feasibility and safety of laparoscopic resection of gastric GISTs (9–12). However, for tumors located in challenging locations, such as the GEJ, lesser curvature, posterior gastric wall and antrum, the difficulty of radical resection of the tumor by laparoscopic surgery is greatly increased. According to the National Comprehensive Cancer Network (NCCN) guidelines (13), laparoscopic surgery is recommended to be performed by experienced surgeons for patients with GISTs in easily accessible anatomical locations. We believe that laparoscopic treatment of gastric gists in challenging anatomical locations has not been recommended mainly because of the flexibility limitations of laparoscopic surgery.

Previous studies have also shown that minimizing the amount of tumor-free tissue resected and maximizing the amount of gastric remnant improve patient quality of life (14). For gastric GISTs located in challenging locations, especially the GEJ or pylorus, surgeons often perform laparoscopic-assisted esophagectomy or subtotal gastrectomy (15, 16), which poses a challenge for preserving gastric function. Currently, the safety and feasibility of robotic surgical resection of gastric GISTs have been demonstrated (17–19). Robot-assisted surgery allows more precise tumor resection and preserves as much gastric tissue as possible. Recent studies have shown that robotic surgery has certain advantages in preserving gastric pylorus and cardia function (20). In this study, we performed robot-assisted surgical endoscopic localization and manually sutured the remnant stomach to treat gastric GISTs in challenging anatomical locations. There is no channel deformity or stenosis caused by excessive resection of tumor-free gastric tissue with this technique.

## Methods

### Patients and study design

In the design of this study, we collected and retrospectively analyzed the clinical data of patients who had undergone robot-assisted surgery combined with upper gastrointestinal endoscopic localization for the treatment of all patients with gastric GISTs located in challenging locations gastric GIST. The data included: The demographic data, clinical manifestations, surgical methods, histopathology, postoperative and oncological outcomes from August 2021 to April 2023. Inclusion criteria: (1) Postoperative pathological diagnosis was gastric stromal tumor. (2) Robot-assisted surgery combined with upper gastrointestinal endoscopic localization was performed. (3) Age ≥18 years old. Exclusion criteria: (1) Gastric stromal tumors located in easily dissected locations. (2) Patients with gastric stromal tumors treated with standard laparoscopic or open surgery. All patients were diagnosed by gastroscopy, EUS or CT before surgery and pathological examination after surgery. All procedures were performed at our center by a single experienced laparoscopic surgeon. All patients provided written informed consent.

### Challenging tumor location definition

According to the National Comprehensive Cancer Network (NCCN) guidelines (13), tumors located in the gastroesophageal junction (GEJ), lesser curvature, posterior gastric wall, or antrum were classified as challenging.

### Surgical procedure

**Figure 1:**
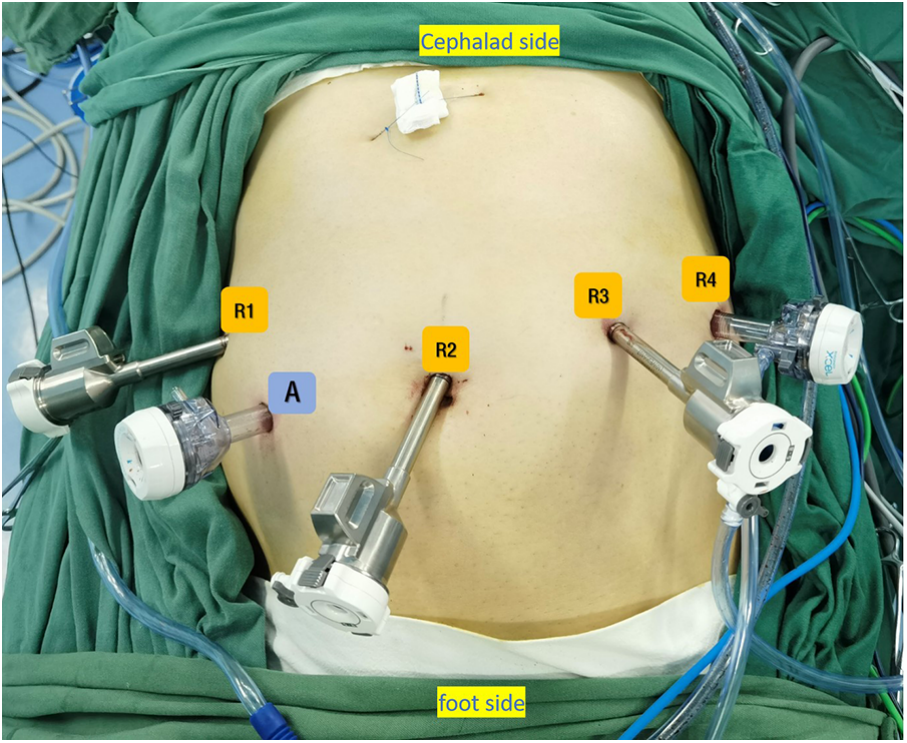
Trocar placement for RALE-PG. (**A**) 12-mm trocar (R2) was placed at the lower umbilical margin, two 8-mm trocars were placed under the costal margin on the left anterior axillary line (R4) and at the intersection of left midclavicular line and a horizontal line 1 cm above the umbilicus (R3), One 8-mm trocar was placed under the costal margin on the right anterior axillary line (R1) and one 12-mm trocar was placed at the intersection of right midclavicular line and a horizontal line 1 cm above the umbilicus (Auxiliary port **A**).

**Figure 2:**
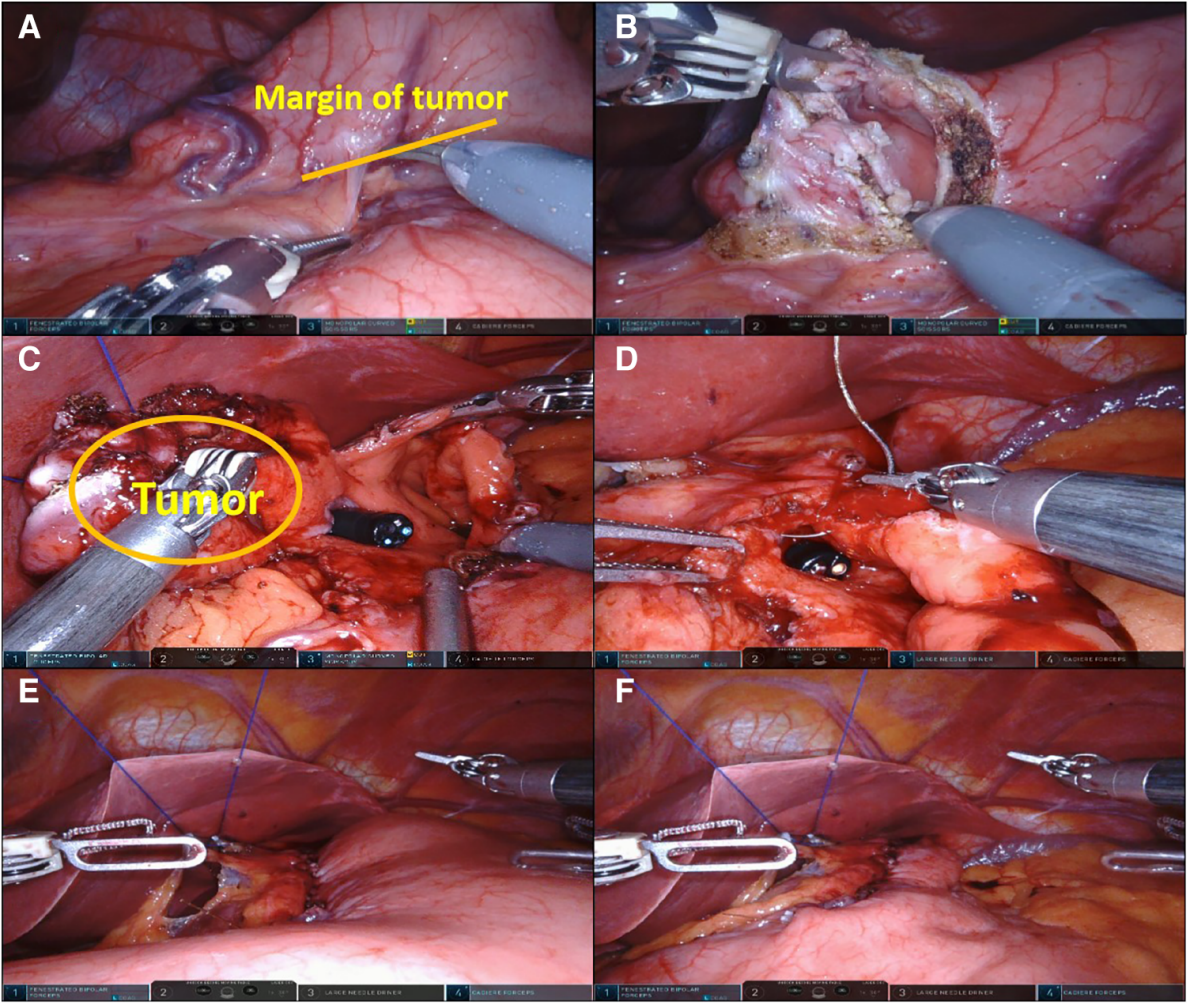
RALE-PG. (**A**) The tumor was localized by upper gastrointestinal endoscopy and robotic laparoscopy; (**B**, **C**). The tumor was accurately removed under the supervision of upper endoscopy; (**D**). Under endoscopic guidance, the remnant stomach was sutured manually; (**E**). Inflation experiments were performed to check for anastomotic condition; (**F**). Key surgical procedures were completed.

After entering the operating room, the patient was placed in the supine position and intubated for anesthesia induction (Figure 1). Upper gastrointestinal endoscopy was routinely performed to provide an intraluminal view of the tumor boundaries to guide precise resection. A parachute suspension technique was used to expose the left subhepatic space (Figure 2). After dissection of the hepatogastric ligaments or gastrocolic ligament, endoscopy was performed to localize the tumor. After removal of the tumor with an ultrasonic scalpel or electric scissors, the gastric wall was repaired by continuous inverted suture with 3-0 barbed suture. Endoscope-guided suturing ensured patency of the gastroesophageal junction or pylorus. The specimen was loaded into a pouch and removed through a periumbilical incision.

### Perioperative management

All patients followed the ERAS protocol as much as possible after performing RALE-PG. Prophylactic intravenous antibiotics (2 g of ceftazidime) were administered half an hour before surgery. A small amount of water was consumed by mouth 12 h after surgery, and when the patient was asymptomatic or could tolerate it, a liquid diet or semiliquid diet was undertaken in sequence. Upper gastrointestinal radiography was performed before removal of the nasogastric tube to assess the function of the residual stomach. At the same time, patients were encouraged to ambulate from bed early to promote the recovery of gastrointestinal function.

### Follow-up

The patients were routinely followed up after surgery by specially trained investigators. Endoscopy was performed in all patients 6–8 weeks after surgery. The last follow-up date for the study was December 2023. The median follow-up time was 19 (10–25) months.

### Statistical analysis

Statistical analysis was performed with SPSS 25.0. Dichotomous data and counts are presented as frequencies, whereas continuous data are presented as X ± SD and/or median (range).

## Results

### Clinicopathologic characteristics

The clinicopathologic characteristics are summarized in Table 1. This study included 12 patients (6 females and 6 males) with a mean age of 56.8 ± 9.8 years and a mean BMI of 23.9 ± 1.9 kg/m^2^. The main clinical manifestations of the patients included abdominal pain/discomfort (*n* = 8, 67%) and no symptoms (*n* = 4, 33%). One of the tumors located in the stomach antrum exhibited exophytic growth, and the other 11 tumors exhibited endoluminal growth. Tumors were located in the GEJ (*n* = 3), lesser curvature (*n* = 3), posterior gastric wall (*n* = 3) and antrum (*n* = 3). The cardia and pylorus were successfully preserved in all patients regardless of the tumor location. The mean tumor size was 4.5 ± 1.4 cm. The mitotic-count/50 mm^2^ was less than 5 in all patients (100%). Pathologic diagnosis was confirmed by immunohistochemistry, revealing gastric GISTs in 12 patients, including 7 at low risk (59%), 4 at moderate risk (33%) and 1 at high risk (8%), according to the Fletcher criteria (21).

**Table 1:** Clinicopathologic characteristics of patients.

Clinicopathologic characteristics	*N* = 12
Sex, male; female, *n* (%)	6 (50%), 6 (50%)
Age (years)	
Mean ± SD; (range)	56.75 ± 9.84; (41–72)
Body mass index (kg/m^2^)	
Mean ± SD; (range)	23.85 ± 1.9; (21.3–26.8)
Comorbidity	
Hypertension, *n* (%)	3 (25%)
Diabetes, *n* (%)	0 (0%)
Heart disease, *n* (%)	0 (0%)
Clinical manifestations	
Abdominal pain/discomfort, *n* (%)	8 (67%)
Asymptomatic, *n* (%)	4 (33%)
Type of growth	
Endophytic, *n* (%)	11 (92%)
Esophytic, *n* (%)	1 (8%)
Tumor location	
GEJ, *n* (%)	3 (25%)
Lesser curvature, *n* (%)	3 (25%)
Posterior gastric wall, *n* (%)	3 (25%)
Antrum, *n* (%)	3 (25%)
Tumor size (cm)	
Mean ± SD; (range)	4.54 ± 1.43; (3.0–8.0)
Mitotic count/5 mm^2^	
≤5 个, *n* (%)	12 (100%)
>5个, *n* (%)	0 (0%)
Risk stratification	
Low, *n* (%)	7 (59%)
Moderate, *n* (%)	4 (33%)
High, *n* (%)	1 (8%)

### Surgical and postoperative outcomes

The surgical and postoperative outcomes are shown in Table 2. Twelve gastric GIST patients underwent upper gastrointestinal endoscopic localization combined with robot-assisted partial gastrectomy. There was no intraoperative tumor rupture (0%) and no conversion to open surgery (0%). The median operation time was 122 (97–240) min, and the median blood loss volume was 10 (5–30) ml. A nasogastric tube was placed in all patients, and an abdominal drainage tube was placed in 6 patients. At 6 h after surgery, the visual analog scale (VAS) score (22) was used to evaluate postoperative pain. The median postoperative VAS score was 2 (2–4). The median time to first flatus was 2 (2–3) days. The median time to first fluid intake was 2 (2–3) days. The median time to first ambulation after the operation was 3 (2–4) days. All patients were routinely examined by postoperative radioscopy before the nasogastric tubes were removed, and no cases of anastomotic stenosis or leakage were found. The median time to drain removal for 6 patients was 5 (4–7) days. The median time to nasogastric tube removal for all patients was 2 (1–5) days. Inflammatory markers (including white blood cell count, procalcitonin and C-reactive protein) were evaluated on the 1st, 3rd, and 5th days after the operation, and we found that the median concentration of inflammatory markers in the blood of patients tended to decrease. The median postoperative hospital stay was 5 (4–8) days. There was no postoperative leakage, abdominal infection, or gastrointestinal dysfunction. One patient (female/41 year) developed moderate anemia (Clavien-Dindo grade II complication), was treated with blood transfusion and was discharged on postoperative day 7. There was no unplanned readmission within 30 days after the operation. The median distance from the tumor to the resection margin was 1 (1–2) cm.

**Table 2:** Surgical and postoperative outcomes of patients.

Surgical outcomes and postoperative courses	*N* = 12
Procedure-related variables	
Type of resection	
Gastrotomy + handsewn suture, *n* (%)	12 (100%)
Conversion to open, *n* (%)	0 (0%)
Tumor rupture, *n* (%)	0 (0%)
Median distance from tumorto margin (cm)	
Median; (range)	1; (1–2)
Operation time (minutes)	
Median; (range)	122; (97–240)
Estimated blood loss(ml)	
Median; (range)	10; (5–30)
Drain	
Yes, *n* (%)	12 (100%)
No, *n* (%)	0 (0%)
Nasogastric tube	
Yes, *n* (%)	6 (50%)
No, *n* (%)	6 (50%)
Post operative functional results	
Postoperative VAS scale	
Median; (range)	2; (2–4)
Time to first flatus (days)	
Median; (range)	2; (2–3)
Time to oral liquid intake (days)	
Median; (range)	2; (2–3)
Time to ambulation (days)	
Median; (range)	3; (2–4)
Gastric radiography	
Stenosis, *n* (%)	0 (0%)
Leakage, *n* (%)	0 (0%)
Time to nasogastric tube remval	
Median; (range)	2; (1–5)
Time to drain remval	
Median; (range)	5; (4–7)
Postoperative white blood cell count(/L)	
Day 1 Median; (range)	9.4; (4.2–13.2)
Day 3 Median; (range)	6.2; (4.6–13.5)
Day 5 Median; (range)	6.0; (4.0–8.6)
Postoperative procalcitonin(ng/ml)	
Day 1 Median; (range)	0.3; (0–30.5)
Day 3 Median; (range)	0.1; (0.1–1.3)
Day 5 Median; (range)	0; (0–0)
Postoperative C-reactive protein(mg/L)	
Day 1 Median; (range)	16.8; (0–157.1)
Day 3 Median; (range)	14.3; (3.2–68.2)
Day 5 Median; (range)	6.3; (1.2–56.2)
Postoperative hospital stay (days)	
Median; (range)	5; (4–8)
Postoperative complications	
Surgical complications	
Yes, *n* (%)	1 (8%)
No, *n* (%)	11 (92%)
Clavien-Dindo	
I–II, *n* (%)	1 (8%)
III–IV, *n* (%)	0 (0%)
Reoperation within 30 days following surgery	
Yes, *n* (%)	0 (0%)
No, *n* (%)	12 (100%)

### Oncologic outcomes

The oncological outcomes are shown in Table 3. R0 resection was achieved in all patients, and the integrity of the mucosal layer was maintained in all patients. The median follow-up period was 19 (10–25) months, and all patients survived with no recurrence or metastasis. Three patients received imatinib as preoperative treatment, and 5 patients received imatinib treatment after surgery (42%).

**Table 3:** Oncologic outcomes of patients.

Oncologic outcomes	*N* = 12
R0 resection, *n* (%)	12 (100%)
Integrity of mucosal layer, *n* (%)	12 (100%)
Negative surgical margins, *n* (%)	12 (100%)
Recurrence, *n* (%)	0 (0%)
Dead for disease, *n* (%)	0 (0%)
Follow-up time (months)	
Median; (range)	19; (10–25)
Imatinib before surgery, *n* (%)	3 (25%)
Imatinib after surgery, *n* (%)	5 (42%)

## Discussion

Our case series focused specifically on gastric function protection in gastric GIST patients with tumors in challenging anatomic locations, including the GEJ (*n* = 3), lesser curvature (*n* = 3), posterior gastric wall (*n* = 3), and antrum (*n* = 3). Compared to patients in the literature, patients in our case series had larger tumors (median size 4.5 cm). In addition, the tumor growth pattern in 11 patients was inward. However, for gastric GISTs in challenging locations, these tumors are not suitable for standard laparoscopic procedures. Based on the experience of Privette (16) and Al-Thani (23), using standard laparoscopic approaches in these areas could lead to excess torque on the lesion and increase the risk of tumor rupture. Because of the unique histopathological features of gastric GISTs, the rate of recurrence in patients with tumor rupture is close to 100% (7, 24). Clearly, RALE-PG overcomes the challenges of standard laparoscopy surgery.

After resection of a gastric stromal tumor in robot-assisted surgery, the remnant gastric wall was sutured by hand instead of using instruments, which benefited from the high flexibility and clear vision of the robotic arm. In terms of surgical safety, there was no conversion to laparotomy or tumor rupture, and our results were better than those of previous studies (RG: 4.2%; LG: 6.3%) (25). Similar to previous studies, no intraoperative tumor rupture occurred in our study [RG: 0% (18); LG: 0% (26)]. The estimated median blood loss volume was 10 (5–30) ml in our study, which is better than that reported in previous studies [RG: 20.0 ml (18); LG: 27.7 ml (11)]. The reason for this may be that the flexible robotic arm and high magnification of the surgical field in robot-assisted surgery allow surgeons to operate more precisely. For the purpose of observing the presence or absence of gastric leakage, abdominal drainage tubes were placed in 6 patients (3 in the GEJ and 3 in the posterior gastric wall), which was a number of patients that was similar to or less than that reported in previous literature (RG: 87.5%; Lg: 53.9%) (25). We prefer to place an abdominal drainage tube in patients with a relatively large remnant gastric anastomosis to observe anastomotic healing and can be used for drainage and decompression if needed. The median operative time in our study was slightly shorter than that in a recent retrospective study (122 vs. 151 min), although it was slightly longer than that in a study with laparoscopic surgery (100 min). This may be related to our experience in performing more than 100 robot-assisted gastrointestinal procedures before we performed this procedure. According to a study by Song et al. (27), the adaptability of robotic surgery increases after 30 operations.

The median postoperative VAS score was 2, and patients experienced only mild postoperative pain. Although few similar studies have focused on these data, in the future, studies with larger sample sizes are needed to confirm these advantages.

The median time to first flatus was 2 days, which was consistent with the findings of a previous study (RG: 2.0 days; LG: 2.0 days). Compared with previous reports (RG: 2.9 days; LG: 3.3 days) (28), the median ambulation time was 3 days, which was not much different. The median time to nasogastric tube removal was 2 days, which was consistent with previous reports (RG: 2 days; LG: 2 days) (25). The median time to abdominal drainage tube removal was 5 days, which was slightly longer than that reported in previous literature (RG: 4 days; LG: 3 days) (25), possibly because of the more conservative drainage tube observation strategy adopted by the surgeon, which generally does not affect the postoperative recovery of patients. Several previous studies have suggested that retaining more gastric tissue is important for gastric function (26) and that traditional gastric wedge resection is more likely to lead to postoperative anastomotic stenosis (14, 25). In our study, manual closure was performed in all patients. Intraoperative upper gastrointestinal endoscopy revealed that the cardia and pylorus were successfully preserved intact without excessive gastric tissue removal. Partial gastrectomy was performed 1 cm from the edge of the tumor, and the gastric wall was repaired with manual sutures. The tissue margin was negative in all patients. Compared with traditional gastric wedge resection, our procedure preserved more normal gastric tissue (1.0 cm vs. 2.5 cm) (20). This suggests that this method has some advantages, but these advantages need to be confirmed in studies with larger sample sizes. All patients underwent upper gastrointestinal radiography before removal of the gastric tube, and no patient experienced stenosis or leakage. A comparative study (25) of different surgical procedures for GIST from Italy showed that robotic surgery appeared to have a lower rate of surgical complications (RG: 4.2%; LG: 6.3%), although there was no statistically significant difference between the groups. All patients underwent upper gastrointestinal endoscopy at 6 months after surgery, and the gastric wall of all patients healed well without ulcers or obvious scar erosion. A report of the modified laparoscopic-endoscopic technique (CLEAN-NET) (26) in Japan showed that there was more irregular scar erosion or ulcers in patients with manual full-thickness sutures. However, this phenomenon did not appear in our study, probably because the greater flexibility and greater lens magnification of the robotic arm in robot-assisted surgery allow the surgeon to easily suture the gastric wall without excessive traction. Of course, the CLEAN-NET has great advantages in preserving gastric function because they only strip the serosa and retain the gastric mucosa and submucosa. However, this technique is difficult to perform for tumors located in challenging anatomical locations, and it is not suitable for tumors that invade the mucosal layer.

Overall, the follow-up period in this study was short, which limited our assessment of long-term oncologic outcomes. Postoperative pathological examination revealed that all patients experienced R0 resection and had negative surgical margins. The blood supply and pseudocapsules of GISTs can easily cause tumor rupture and implantation metastasis during surgery. Therefore, whether R0 resection had been achieved had a great impact on patient prognosis. No recurrence occurred in this cohort at a median follow-up of 19 months. Our results are similar to or better than those of previous studies [100% vs. 95% (29), 100% vs. 87% (24)].

Our study demonstrated the safety and efficacy of this procedure and its advantages in preserving gastric function. However, our study has several limitations. First, the sample size was small, the study was retrospective, and there was no control group. Second, patients included in the present study were mostly assessed as having a low risk of tumor recurrence, which may have biased the assessment of medium- and long-term survival. Finally, the follow-up period in our study was relatively short, so a longer follow-up period is needed to assess the true value and long-term outcome of this technique.

## Conclusions

RALE-PG is a safe, feasible and advantageous technique for treating GISTs in challenging anatomical locations. It can be performed to accurately remove the tumor while preserving gastric function to the greatest extent. None of the patients in this study experienced surgical complications or tumor recurrence during the follow-up period, but oncologic outcomes need to be evaluated in studies with a larger sample size and longer follow-up period.

## Data Availability

The original contributions presented in the study are included in the article/Supplementary Material, further inquiries can be directed to the corresponding authors.
